# Video prediction based on temporal aggregation and recurrent propagation for surveillance videos

**DOI:** 10.1016/j.mex.2025.103402

**Published:** 2025-06-06

**Authors:** Mohana Priya P, UlagaPriya K

**Affiliations:** Department of Computer Science & Engineering, Vels Institute of Science, Technology & Advanced Studies(VISTAS), Chennai, India

**Keywords:** Prediction, Frames, Inpainting, Interpolation, Temporal aggregation, Recurrent propagation, Time steps, Bidirectional Video Prediction Network

## Abstract

Video prediction is essential for recreating absent frames in video sequences while maintaining temporal and spatial coherence. This procedure, known as video inpainting, seeks to reconstruct missing segments by utilizing data from available frames. Frame interpolation, a fundamental component of this methodology, detects and produces intermediary frames between input sequences. The suggested methodology presents a Bidirectional Video Prediction Network (BVPN) for precisely forecasting absent frames that occur before, after, or between specified input frames. The BVPN framework incorporates temporal aggregation and recurrent propagation to improve forecast accuracy. Temporal aggregation employs a series of reference frames to generate absent content by harnessing existing spatial and temporal data, hence assuring seamless coherence. Recurrent propagation enhances temporal consistency by integrating pertinent information from prior time steps to progressively improve predictions. The timing of frames is constantly controlled through intermediate activations in the BVPN, allowing for accurate synchronization and improved temporal alignment. A fusion module integrates intermediate interpretations to generate cohesive final outputs. Experimental assessments indicate that the suggested method surpasses current state-of-the-art techniques in video inpainting and prediction, attaining enhanced smoothness and precision. Surveillance video datasets demonstrate substantial enhancements in predictive accuracy, highlighting the strength and efficacy of the suggested strategy in practical application.

• The proposed method integrates bidirectional video prediction, temporal aggregation, and recurrent propagation to effectively reconstruct missing intermediate video frames with enhanced accuracy.
• Comparative analysis using the UCF-Crime dataset demonstrates higher PSNR and SSIM values for the proposed method, indicating improved frame quality and temporal consistency over existing techniques.
• This research provides a robust framework for future advancements in video frame prediction, contributing to applications in anomaly detection, surveillance, and video restoration.

## Graphical abstract

**Figure:**
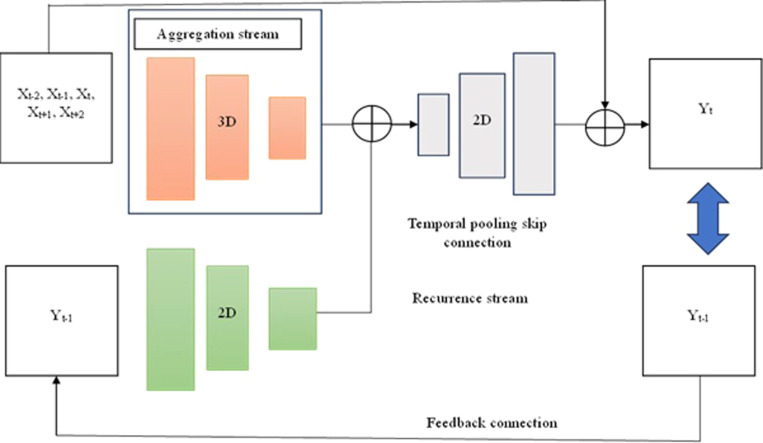

Specifications table

**Table:** 

Subject area:	Computer Science
More specific subject area:	Deep Learning
Name of your method:	Bidirectional Video Prediction Network
Name and reference of original method:	If applicable, list the full bibliographic details of any key reference(s)that describe the original method you customized
Resource availability:	• P. Hu, J. Wu, Z. Yan, M. He, C. Liang, H. Bai, Warhead fragments motion trajectories tracking and spatio-temporal distribution reconstruction method based on high-speed stereo photography, Def. Technol. 37 (2024) 162–172. https://doi.org/10.1016/J.DT.2024.02.009. • S. Sengupta, S. Basak, P. Saikia, S. Paul, V. Tsalavoutis, F. Atiah, V. Ravi, A. Peters, A review of deep learning with special emphasis on architectures, applications and recent trends, Knowledge-Based Syst. 194 (2020) 105,596. https://doi.org/10.1016/J.KNOSYS.2020.105596. • E. Dhamala, L.Q. Rong Ooi, J. Chen, J.A. Ricard, E. Berkeley, S. Chopra, Y. Qu, X.H. Zhang, C. Lawhead, B.T.T. Yeo, A.J. Holmes, Brain-Based Predictions of Psychiatric Illness–Linked Behaviors Across the Sexes, Biol. Psychiatry. 94 (2023) 479–491. https://doi.org/10.1016/J.BIOPSYCH.2023.03.025. • H.J. Lee, A. Dworetsky, N. Labora, C. Gratton, Using precision approaches to improve brain-behavior prediction, Trends Cogn. Sci. (2024). https://doi.org/10.1016/J.TICS.2024.09.007. • J. Luo, J. Zhang, A Method for Image Anomaly Detection Based on Distillation and Reconstruction, Sensors. 23 (2023) 9281. https://doi.org/10.3390/S23229281. • E.M. Mercha, H. Benbrahim, Machine learning and deep learning for sentiment analysis across languages: A survey, Neurocomputing. 531 (2023) 195–216. https://doi.org/10.1016/J.NEUCOM.2023.02.015. • D. Oosterlinck, D.F. Benoit, P. Baecke, From one-class to two-class classification by incorporating expert knowledge: Novelty detection in human behaviour, Eur. J. Oper. Res. 282 (2020) 1011–1024. https://doi.org/10.1016/J.EJOR.2019.10.015.

## Background

The extensive implementation of video surveillance systems is essential for guaranteeing public safety and security, especially in the real-time monitoring and identification of anomalous behaviour. The gradual implementation of these technologies has led to an exponential increase in the volume of video data produced for societal surveillance [1,2]. Manually analysing this extensive data set to detect suspicious activity is inefficient, time-consuming, and susceptible to human error. This topic has generated considerable interest in the creation of automated algorithms that can identify odd behaviours in surveillance film. Nevertheless, delineating and recognizing anomalies in surveillance footage is a challenging endeavour due to their intrinsically ambiguous characteristics and absence of clearly defined parameters [3]. The rare occurrence of abnormal occurrences complicates this approach, as obtaining representative anomalous samples from large datasets is a significant problem. Conversely, acquiring standard examples from surveillance footage is rather simple, establishing a basis for algorithm development [4]. Semi-supervised learning approaches have developed as a viable solution to the scarcity of aberrant data in training sets. These methods primarily utilize normal data to train models that can identify deviations suggestive of abnormalities, therefore improving the efficiency and reliability of anomaly detection in video surveillance systems.

Anomaly identification in video surveillance often entails examining regular patterns within training data and observing their temporal progression. Anomalies, characterized as deviations from standard patterns, are recognized upon their occurrence. Semi-supervised anomaly detection techniques are primarily classified into prediction-based and reconstruction-based methods, both of which are essential in the domain [5]. Historically, reconstruction-based methodologies depended on hand crafted appearance and motion attributes. These systems frequently employed dictionary learning to represent common events and encode them with minimal error. Anomalies in testing were deduced from substantial reconstruction mistakes of certain features [6]. Nonetheless, dictionary learning is constrained by its dependence on manually designed features and the computationally demanding task of calculating sparse coefficients, which diminishes efficiency and scalability for extensive datasets. The emergence of deep learning has revolutionized this paradigm, facilitating the automatic extraction of intricate features through deep neural networks, hence obviating the need for manual feature engineering [7]. Autoencoders have been utilized for the precise reconstruction of normal events, capitalizing on their capacity to generalize effectively to standard data while emphasizing anomalies in atypical frames. Deep neural networks excel in this field owing to their resilience and generalization abilities. Prediction-based methods have arisen as a supplementary approach, concentrating on simulating future normative events. These techniques seek to forecast subsequent frames in a series; substantial prediction errors signify the existence of anomalies, as unforeseen events disturb temporal coherence [8]. The suggested video prediction algorithm utilizes a Fine-tuned Frame Predictor (FFP) to improve anomaly detection. The FFP employs a U-Net architecture to forecast future frames by examining temporal and spatial data from prior frames. A substantial divergence between the anticipated and actual frames signifies anomalous occurrences, offering a dependable method for anomaly identification in video surveillance systems [9].

The proposed method demonstrates effective performance in anomaly detection when assessed on publically available datasets, accurately recognizing deviations from standard patterns [10]. Nonetheless, prediction-based methodologies encounter difficulties in consistently attaining minimal prediction errors for routine occurrences, notwithstanding their notable performance on benchmark datasets. Video Anomaly Detection (VAD) is a crucial element for the effective operation of video surveillance systems, as it facilitates the detection of atypical events vital for ensuring public safety [11]. A significant obstacle in VAD is the lack of aberrant data samples necessary for the proper training of machine learning models. This constraint frequently requires reinterpreting the issue as a one-class classification problem. This method involves the model concentrating on understanding the distribution of normal data to identify anomalies, depending solely on the statistical properties of typical occurrences [12]. This simplification facilitates the reliable identification of anomalies by modeling standard distances, even without substantial aberrant training data, thus improving the system's efficiency and relevance in practical situations.

Variational Autoencoder (VAE) techniques have proven to be efficient for anomaly detection, especially in cases where a model inadequately reconstructs or predicts particular data samples. These methods utilize the premise that normal data samples closely conform to a learnt normal distribution, leading to negligible reconstruction or prediction errors during inference. In contrast, anomalies demonstrate markedly greater errors owing to their divergence from the established normal distribution [13]. For this assumption to be valid, the model must have the representational capacity to accurately reflect the fundamental characteristics of normal data. Modeling intricate and high-dimensional data, such as video, has distinct issues. Effectively capturing the essential elements of video footage, such as visual characteristics, dynamic movements, and temporal sequences, necessitates sophisticated approaches capable of concurrently addressing these interconnected dimensions [14]. We propose a Bidirectional Video Prediction Network (BVPN) to accurately forecast missing frames in surveillance movies, addressing these problems. The BVPN framework utilizes temporal aggregation and recurrent propagation to harness information from prior and subsequent frames. Through the reutilization and synthesis of temporal data, the network attains precise inpainting of absent frames, guaranteeing temporal coherence and augmenting anomaly detection skills in video surveillance systems.

### Video anomaly detection methods

#### Reconstruction

Training methods for video anomaly identification seek to simulate the typical distribution of video data to facilitate high-quality reconstructions. A substantial reconstruction error during inference signifies a divergence from the acquired normal distribution, implying the existence of anomalies [15]. Convolutional autoencoders have been extensively advocated for reconstructing input frames due to their versatility and efficacy in capturing spatial and temporal characteristics. Recent improvements have investigated multiple extensions of convolutional autoencoders, such as parametric density estimators, memory-augmented autoencoders, and two-stream recurrent frameworks, to improve performance [16]. Reconstruction-based methods emphasize the reconstruction of frames from the foundation, utilizing the encoded understanding of standard patterns. Nonetheless, these methodologies encounter significant obstacles, including the failure to adequately reconstruct abnormal occurrences, precisely distinguish between normal and anomalous data, and alleviate overfitting. These constraints underscore the necessity for more resilient architectures that can tackle the intrinsic complexity of video anomaly detection while ensuring generalization and scalability.

#### Prediction

Anomaly identification in movies often use prediction-based methods, where future frames are forecasted based on the temporal information of prior frames. The fundamental concept asserts that predictable events conform to established patterns, whereas anomalies diverge unpredictably from these standards [17]. Prior research has established a basis for standardizing prediction tasks through the integration of consistency metrics. Venkatesh et al. [18] Introduced a method that utilizes gradient and intensity limitations to enhance the precision of frequent event predictions. This method standardizes projected outcomes by juxtaposing pixel values of the forecasted frames with their actual equivalents, hence improving prediction accuracy. Alongside conventional single-modality restrictions, modern methodologies have investigated the amalgamation of intricate modality memory pools to enhance the representation of the coherence between appearance and motion data [19]. By integrating characteristics from many modalities, these approaches produce a more resilient representation of predominant occurrences, enhancing anomaly identification and predictive accuracy. A unique prediction paradigm emphasizes utilizing temporal information in movies to recover omitted segments of events removed from the sequence [20]. This approach frequently overlooks the complex link between optical flow and video frames, depending exclusively on pixel-wise limitations. In contrast to conventional methods that focus on individual video anomalies, contemporary prediction tasks seek to evaluate and derive extensive insights from video data as a whole, regardless of its temporal sequence or format [21]. This transition highlights the increasing emphasis on optimizing the use of all accessible information to improve anomaly detection and predictive abilities.

### Traditional methods used in anomaly detection

Historically, feature spaces for anomaly detection have been created using either manually crafted features or features derived from conventional machine learning techniques. These methods utilize domain-specific expertise or statistical tools to discern patterns in the data [22]. Subsequent to feature generation, the distributions of normal and anomalous cases are examined to locate outliers or identify clusters that significantly diverge from standard patterns within the dataset [23]. Although these techniques are fundamental, they frequently encounter constraints in representing intricate, high-dimensional relationships present in video data, prompting the need for the investigation of more sophisticated and automated feature extraction methods.

### Statistical model methods

The dynamic trajectory features of objects are frequently utilized as a fundamental method in research that employs statistical models to illustrate typical motion patterns. This mechanism has proven crucial in independently acquiring motion behaviours. A significant work introduced a technique for anomaly identification by online trajectory clustering, which combines clustering with data acquisition via a tracking system. Nonetheless, the trajectory aspects of object tracking exhibit diminished dependability in intricate settings, especially in scenarios characterized by high object density or occlusions, resulting in performance decline [24]. To tackle these issues, low-level spatiotemporal characteristics have arisen as a formidable substitute for trajectory-based features in improving anomaly identification. Methods like Histograms of Oriented Gradients (HOGs) and Histograms of Oriented Flows (HOFs) have been extensively utilized to represent spatiotemporal features. Wang et al. [25] investigated the application of low-level spatiotemporal characteristics in conjunction with local histograms of light fluxes across diverse spatial regions. The data were analysed utilizing a Gaussian Mixture Model (GMM), subsequently enhanced by the integration of Mixture of Dynamic Textures (MDTs) [26], thus augmenting anomaly identification in intricate and dynamic video contexts.

### Sparse coding methods

Initial methodologies employing sparse coding for anomaly identification depended on the acquisition of dictionaries based on manually crafted characteristics. These approaches sought to precisely reconstruct normal events with minimal inaccuracies, classifying occurrences with substantial reconstruction mistakes as aberrant [27]. Sparse Reconstruction Cost (SRC) was established as a criterion to assess the congruence of test samples with a specified standard dictionary. An unsupervised dynamic sparse coding method was developed to improve anomaly detection, facilitating the identification of atypical events in movies by online sparse reconstruction of query signals using an atomically learnt event dictionary [28]. Additionally, an efficient Sparse Combination Learning (SCL) framework was developed to tackle the computing difficulties linked to optimizing sparse coefficients, hence expediting testing and training procedures [29]. Although conventional sparse coding methods have demonstrated efficacy in particular contexts, they encounter difficulties in complex situations owing to their restricted capacity to encode complicated aspects. Moreover, the computational complexity of these traditional methods frequently obstructs real-time anomaly identification in movies, hence complicating their practical implementation [30].

### Deep learning-methods

Methods based on deep learning have exhibited exceptional efficacy in multiple fields, such as picture classification, object identification, and video retrieval. These achievements have facilitated various deep learning methodologies for video anomaly detection [31]. These techniques are generally classified into prediction-based and reconstruction-based methods. Reconstruction-based methods have substantial parallels to sparse coding techniques; however, recent developments utilizing deep features in reconstruction have markedly surpassed previous methods dependent on hand-crafted features [32]. A two-stream neural network was presented to improve the extraction of spatial-temporal fusion features (STFF), enabling a strong integration of spatial and temporal data. The STFF was actively utilized alongside a fast sparse coding network (FSCN) to produce a standard dictionary in real-time. An autoencoder, trained on fully connected neural networks, was employed to decode temporal patterns in video data, utilizing extracted features as input [33]. Irregularities were detected by calculating a regularity score based on reconstruction errors. Nevertheless, the constraints of 2D convolution rendered motion data extraction unachievable within this framework, underscoring a potential area for improvement [34].

Deep neural networks demonstrate negligible variations in reconstruction error rates when differentiating between typical and atypical events. This problem stems from their substantial capacity, generalizability, and sophisticated feature extraction skills. To mitigate this constraint, researchers have suggested diminishing the capacity of convolutional neural networks (CNNs) for data encoding, supplemented by the incorporation of a memory module to retain archetypal input patterns [35]. Recent years have witnessed substantial advancements in video prediction methodologies, utilizing extensive quantities of unlabelled data to acquire internal video representations. These strategies possess several applications, encompassing video comprehension, autonomous driving, and robotic decision-making. Video prediction entails anticipating subsequent frames by the analysis of prior ones [36]. An adversarial training multi-scale network was presented to improve frame forecasting, facilitating the production of future frames given an input sequence. Yin et al. [37] developed a recurrent autoencoder utilizing long short-term memory (LSTM) networks to record temporal correlations among patches derived from sequential input frames, aimed at video forgery detection. These video prediction frameworks have a notable capacity to recognize routine events. Nevertheless, when confronted with infrequent and unpredictable events, frequently classified as anomalies, their precision declines, highlighting the necessity for more enhancements in anomaly detection [38].

The primary application of video prediction was in visual anomaly detection, founded on the notion that prediction errors could signify irregularities. A Long Short-Term Memory (LSTM) model with convolutional feature representations was developed to forecast mistakes, which were then used to detect anomalies in robotics applications. A Conv-LSTM network, an end-to-end trainable composite architecture, was proposed to predict the future trajectory of video sequences in various contexts [39]. Expanding upon this basis, an alternative method utilized the U-Net architecture to forecast anomalies in videos through predictive analysis. This approach integrated adversarial loss, optical flow, and the discrepancy between expected and actual frames to enhance network performance for superior anomaly identification. A predictive coding network was presented, incorporating an error refinement module and a predictive coding module to improve anomaly detection skills. A novel method integrates forward, backward and retrospective prediction techniques to comprehensively investigate bidirectional mapping relationships in video frame sequences. The implementation of a three-dimensional convolutional neural network (3D-CNN) sequence discriminator improved the temporal consistency of anticipated frames, hence augmenting anomaly detection efficacy. The amalgamation of predictive coding and sophisticated neural architectures represents a notable progression in visual anomaly detection techniques

## Method details

The suggested methodology seeks to reconstruct absent video sequences by utilizing information from prior and subsequent frames to attain seamless and visually cohesive outcomes through sophisticated video inpainting techniques. A deep neural network architecture is presented to optimize and approximate the intricate functions associated with video inpainting. In contrast to traditional methods that directly correlate the sequence of prior and subsequent frames to the missing segment, the suggested methodology utilizes a modular architecture, partitioning the issue into two separate elements: a bidirectional video prediction network and a temporal aggregation module. The bidirectional prediction network records temporal relationships between consecutive frame sequences, while the temporal aggregation module employs recurrent propagation core functions to consolidate temporal information and maintain continuity. This systematic and modular methodology improves the accuracy and realism of the reconstructed video sequences, exceeding the constraints of conventional approaches.

### Architecture of the proposed system

**Fig. 1:**
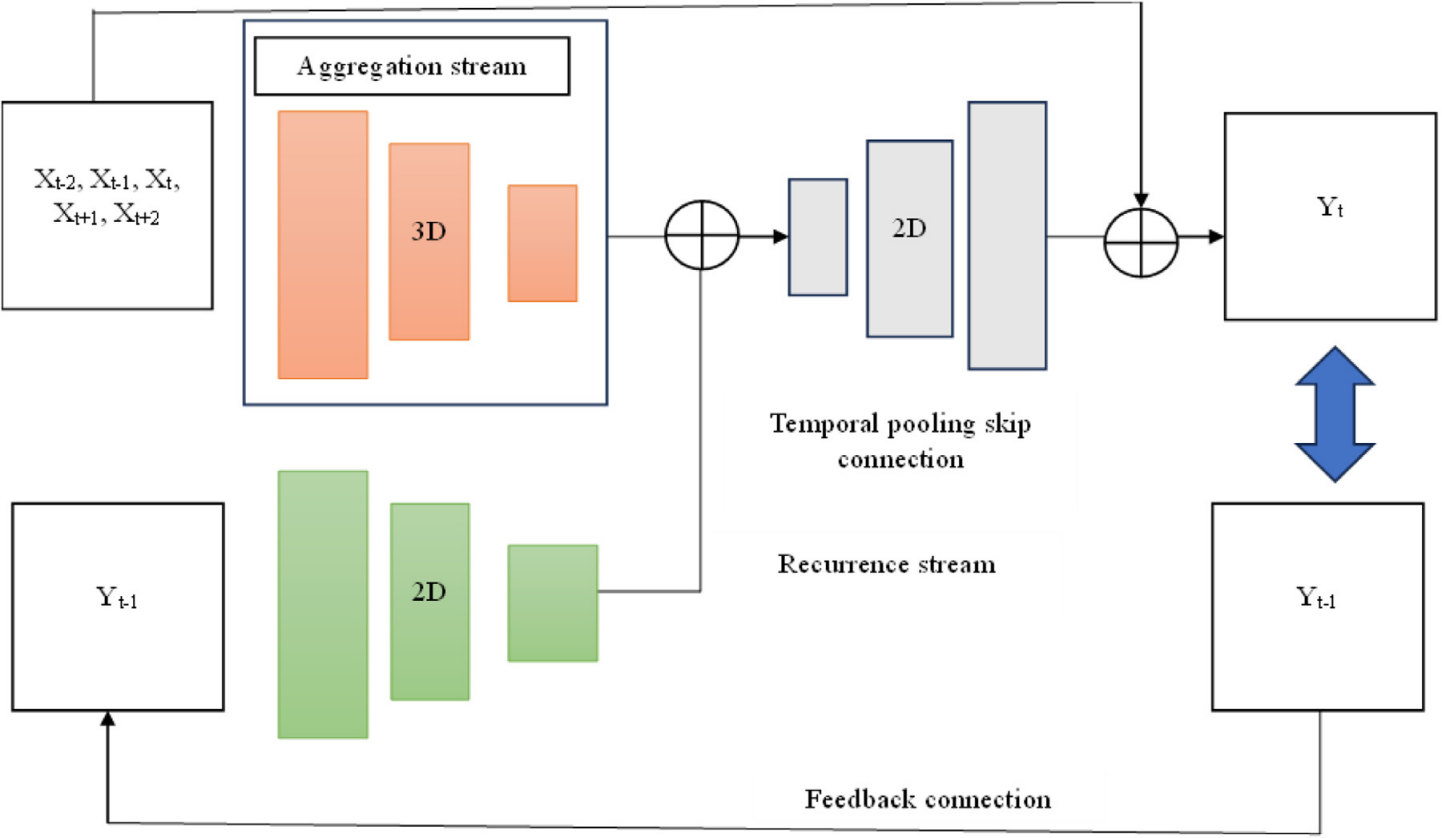
Architecture of the proposed system.

Fig. 1 depicts the architecture of the proposed system, engineered for efficient video frame reconstruction. The procedure commences with the identification of a target frame from a video clip, which acts as input for the temporal aggregation module. This module consists of three essential components: an encoder, an aggregator, and a decoder. The encoder converts the target frame into a binary format, enabling computer processing. The temporal aggregation methods utilized encompass average calculation, cluster creation, and heuristic strategies. Average determination is employed for video downsampling by computing temporal averages to minimize redundancy while maintaining critical information. Cluster creation categorizes temporal slices into separate groups within a multidimensional space, with each group defined by the average of its time steps. This approach accepts varying durations, offering flexibility and adaptability in processing varied video inputs.

The suggested recurrent system combines temporal aggregation and feedback methods to rebuild video frames. The encoder, upon receiving input from the prior phase, recognizes the target image and processes the impaired area using three-dimensional convolution operations. The encoder's output is transmitted to the aggregator, which does an element-wise summation to combine temporal characteristics. The combined output functions as input for the decoder, which utilizes two-dimensional convolution to recreate the frame. The reconstructed frame is evaluated against the original reference frame to determine correctness. Subsequently, a deception frame is presented to the decoder, and its output is relayed back to the aggregator, facilitating iterative enhancement via temporal aggregation and recurrent propagation. This cyclical method guarantees the efficient restoration of absent or impaired video parts.

#### Temporal aggregation

The bidirectional video prediction network aims to approximate and reconstruct intermediate sequences by utilizing the temporal coherence of prior and subsequent frames. This network utilizes sampled frames as reference points to deduce and finish the target frame, emphasizing visible parts within the sequence to maintain continuity and structural integrity. The framework improves the precision of sequence approximation and effectively reconstructs intermediate frames by synthesizing information in both directions, maintaining temporal and spatial consistency.

#### Recurrent propagation

Temporal consistency is attained by efficiently utilizing existing spatiotemporal data, in which the temporal coordinates of individual frames are recorded via intermediate activations inside the bidirectional video prediction network. This network combines temporal advancement with improved time intervals to guarantee a coherent sequence reconstruction. The frames processed by the bidirectional video prediction network are enhanced and unified using a fusion module that dynamically integrates spatial and temporal data to preserve structural and contextual coherence across successive frames.

#### Bidirectional video prediction network

Two distinct varieties of middle predictions are produced by bidirectional video prediction. They are denoted by Nu and Gu. Nu denotes the middle sequence and depends on the prior sequence. Gu denotes the following sequence.

The prediction in the forward direction is given by

(1) $${\hat{N}}_{U}^{Q}=\left\{U_{q+1}^{Q},........U_{q+m}^{Q}\right\}$$

The prediction in the reverse direction is given by

(2) $${\hat{N}}_{U}^{Q}=\left\{U_{q+1}^{Q},........U_{q+m}^{Q}\right\}$$

By restricting the forward and backward sequences with conditions, we obtain

(3) $${\hat{N}}_{U}^{Q}=\theta_{prediction}\left(Q_{u}\right)$$

(4) $${\hat{N}}_{U}^{Q}=\theta_{prediction}\left(Q_{u}\right)$$

R denotes the backward operation function. The same parametric values are used for the prediction of the forward and backward interpretations. The bidirectional video prediction network generates one frame sequentially. The prior frames are conditioned during the frame production. During forward interpretation, ${\tilde{u}}_{t1}^{Q}$ will be ut if t belongs to {1….q} or ${\tilde{u}}_{t1}^{Q}$ if ut belongs to {*q* + 1……*q* + *n*}. The inputs to the interpolation network are the activations saved from the bidirectional video prediction network.

(5) $${\hat{u}}_{L+1}^{Q}=\theta_{prediction}\left\{\tilde{u}Q_{1},\tilde{u}Q_{2},\tilde{u}Q_{3},.......\tilde{u}Q_{L}\right\}$$

#### Frame interpolation and temporal aggregation

This technique blends the frames provided by the bidirectional video prediction network. The final prediction is produced after this step. The middle activations are leveraged and the temporal space are denoted by the enhanced time steps.

Frame interpolation produces the final frame by combining $\hat{{N_{u}}^{Q}}$ and $\hat{{N_{u}}^{Q}}$. It uses the same time step. Combining $\hat{{u_{u}}^{Q}}$ and $\hat{{u_{u}}^{Q}}$ is tedious because there are still problems that do not match. Another reason for the combination difficulty is that they are not reliable.

(6) $${\hat{u}}_{T}=\theta_{blend}\left({\hat{u}}_{T}^{Q},{\hat{u}}_{T}^{G}\right)$$

(7) $${\hat{u}}_{T}=\theta_{blend}\left({\hat{u}}_{T}^{Q},{\hat{u}}_{T}^{G}\right)$$

For combining the frames in a more accurate manner, the proposed method uses two other data. Apart from the frames to be combined, the interpretation is performed based on the received time steps and the activations received from the bidirectional video prediction network.

(8) $${\hat{u}}_{T}=\theta_{blend}\left({\hat{u}}_{T}^{Q},{\hat{u}}_{T}^{G}\right)$$

(9) $${\hat{u}}_{T}=(\theta_{temp\text{int}erpolation}\left({{\hat{u}}_{T}}^{Q},{{\hat{f}}_{T}}^{Q},{{\hat{u}}_{T}}^{G},{{\hat{f}}_{T}}^{G},w_{T}\right)$$

In temporal aggregation, a two-dimensional kernel is applied to every area. The final image can be obtained by adding the pixels. A model based on an encoder and decoder is used for the production of these adjustable kernels. ${{\hat{f}}_{T}}^{Q}$ and ${{\hat{f}}_{T}}^{Q}$ are the enhancement steps of time. ${L_{T}}^{Q},{L_{T}}^{G}$corresponds to how high and wide the resolution of the frame is. The enhancement time step is scaled into one of the decoder’s hidden layer outputs. The adjustable kernels are applied to the inputs and are added by means of the pixels to determine the final image.

(10) $${L_{T}}^{Q},{L_{T}}^{G}={\theta_{blend}}^{encoderdecoder}\left({{\hat{u}}_{T}}^{F},{{\hat{f}}_{T}}^{F},w_{T}\right)$$

(11) $$\hat{u}\left(a,b\right)={L_{T}}^{Q}\left(a,b\right)*Qq\left(a,b\right)+{L_{T}}^{G}\left(A,b\right)*P_{G}\left(a,b\right)$$

## Method validation

### Dataset and training details

The suggested prediction method was executed in Python 3, utilizing the PyTorch and OpenCV libraries for deep learning model training and image processing. Experiments utilized the UCF-Crime dataset, an extensive compilation of authentic surveillance footage, consisting of 1900 unedited movies across 13 distinct categories of abnormal events. This dataset has 1610 training videos and 180 test videos. The proposed model was assessed by categorizing video clips from both the dataset and those generated by the model using a binary classification method based on convolutional neural networks (CNNs). The bidirectional video prediction network (BVPN) underwent training for 10,000 epochs with a batch size of 32, utilizing the Adam optimizer to provide effective feature extraction and precise anomaly identification.

### Technical details

**Fig. 2:**
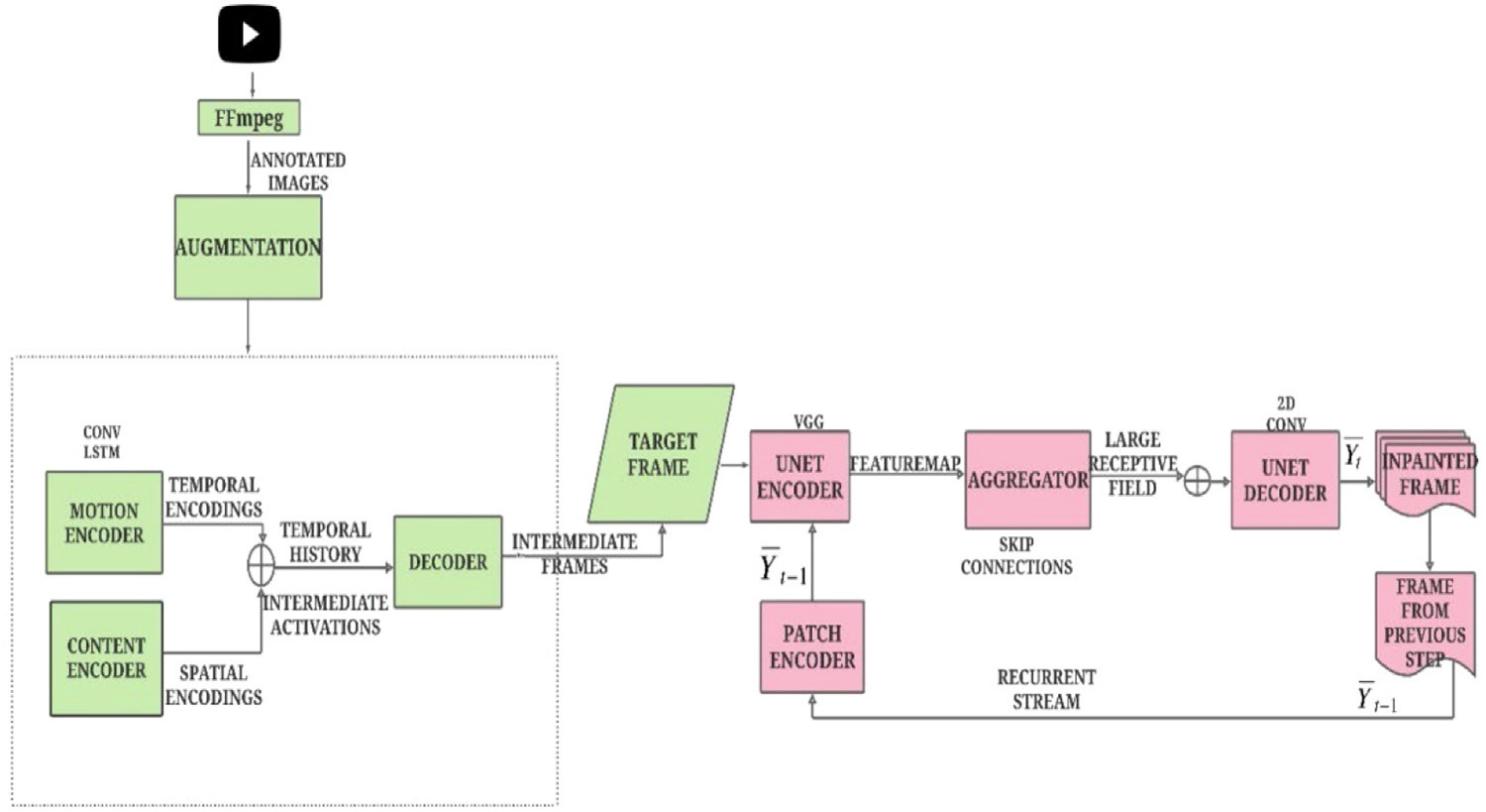
Network details of BVPN.

The video frame inpainting model employs trained reconstruction and adversarial objective functions to improve interpretative precision. Fig. 2 illustrates the bidirectional video prediction network, wherein video sequences are initially transformed into individual frames via FFmpeg, succeeded by picture augmentation to improve resilience. A motion control network is utilized to examine spatial and temporal encodings, facilitating the recognition of temporal history via intermediate activations and scaled time intervals. The intermediate frames are then inpainted via temporal aggregation, enabled by a U-Net encoder-decoder architecture. The recurrent stream is facilitated by a patch encoder functioning as a discriminator, ensuring precise reconstruction and preserving temporal consistency throughout the video frames.

(12) $$L_{h}=\alpha [L_{image}\left({{\hat{N}}_{u}}^{Q},N_{u}\right)+L_{image}\left({{\hat{N}}_{u}}^{G},N_{u}\right)+L_{image}\left({\hat{N}}_{u},N_{u}\right)+\beta L_{adv\_loss}\left({\hat{N}}_{u}\right)$$

(13) $$L_{adv\_loss}\left(\hat{N_{u}}\right)=-\log D\left(\left[Q_{u},\hat{N_{u}},F_{u}\right]\right)$$

(14) $$L_{image}\left({\hat{N_{u}}}^{\left(.\right)},N_{u}\right)=L_{2}\left({\hat{N_{u}}}^{\left(.\right)},N_{u}\right)+L_{grd\_diff\_loss}\left({\hat{N_{u}}}^{\left(.\right)},N_{u}\right)$$

The generator and discriminator are updated one by one in an alternating fashion. The structure loss can be minimized by using the following expression

(15) $$L_{2}\left({\hat{N_{u}}}^{\left(.\right)},N_{u}\right){=|}_{2}^{2}$$

$$L_{grd_{diff_{loss}}}\left({N_{u}}^{\left(.\right)},N_{u}\right)=\sum_{T=Q+1}^{Q+n}\sum_{j,i}^{w,h}(\left|u_{T}\left(j,i\right)-u_{T}\left(j-1,i\right)\right|-|{u_{T}}^{\left(.\right)}\left(j,i\right)-{u_{T}}^{\left(.\right)}\left(j-1,i\right)\left|\right)+$$

(16) $$\left(\left|u_{T}\left(j,i-1\right)-u_{T}\left(j,i-1\right)\right|\right)-{u_{T}}^{\left(.\right)}\left(j,i\right)-{u_{T}}^{\left(.\right)}\left(j,i-1\right)\left|\right)$$

In the aforementioned formula, alpha and beta represent the reconstructive loss and the combative loss, respectively. The intermediate interpretations and the resultant interpretations are concurrently tracked here. The Limage comprises the squared mismatch error and the gradient difference mismatch. The precision in the peripheries of the images can be enhanced by detecting and correcting these deficiencies.

**Fig. 3:**
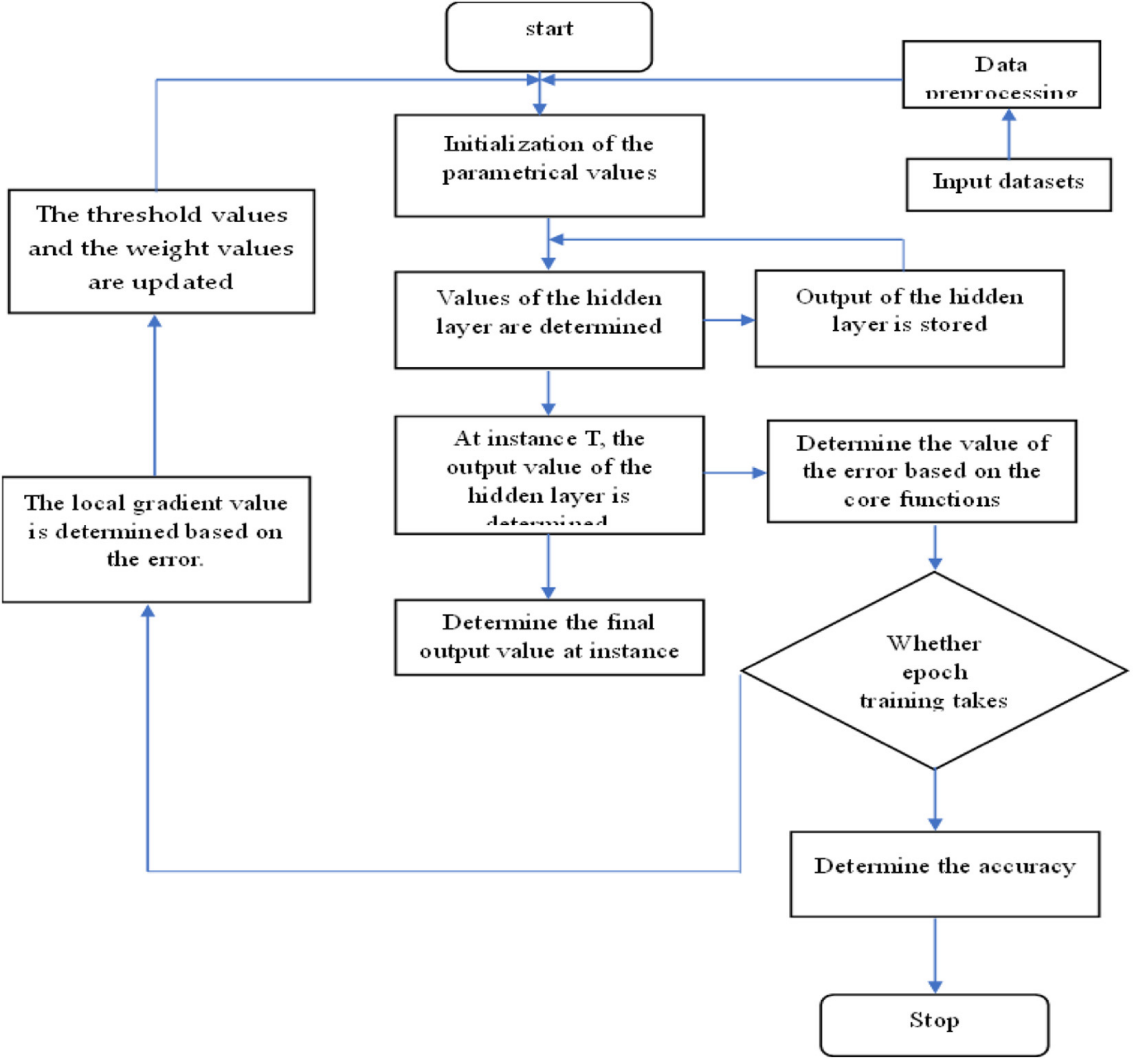
Flow diagram of the framework with the two core functions.

Fig. 3 depicts the flow diagram of the proposed framework, which functions based on two fundamental operations. The procedure commences with the initialization of parameters, succeeded by the initialization of hidden layer values. The pre-processed data are subsequently entered into the network to calculate the values of the hidden layer. At time T, the output from the hidden layers is ascertained and preserved for subsequent processing. The inaccuracy is subsequently computed based on the fundamental functions. The output value at time T is subsequently calculated. The subsequent phase entails ascertaining whether epoch training has been finalized. Upon completion of training, precision is computed, and the procedure concludes. If training is incomplete, the local gradient value is calculated based on the error, followed by the updating of weights and thresholds. This cycle persists with the re-initialization of the hidden layer values, guaranteeing the iterative enhancement of the model.

**Fig. 4:**
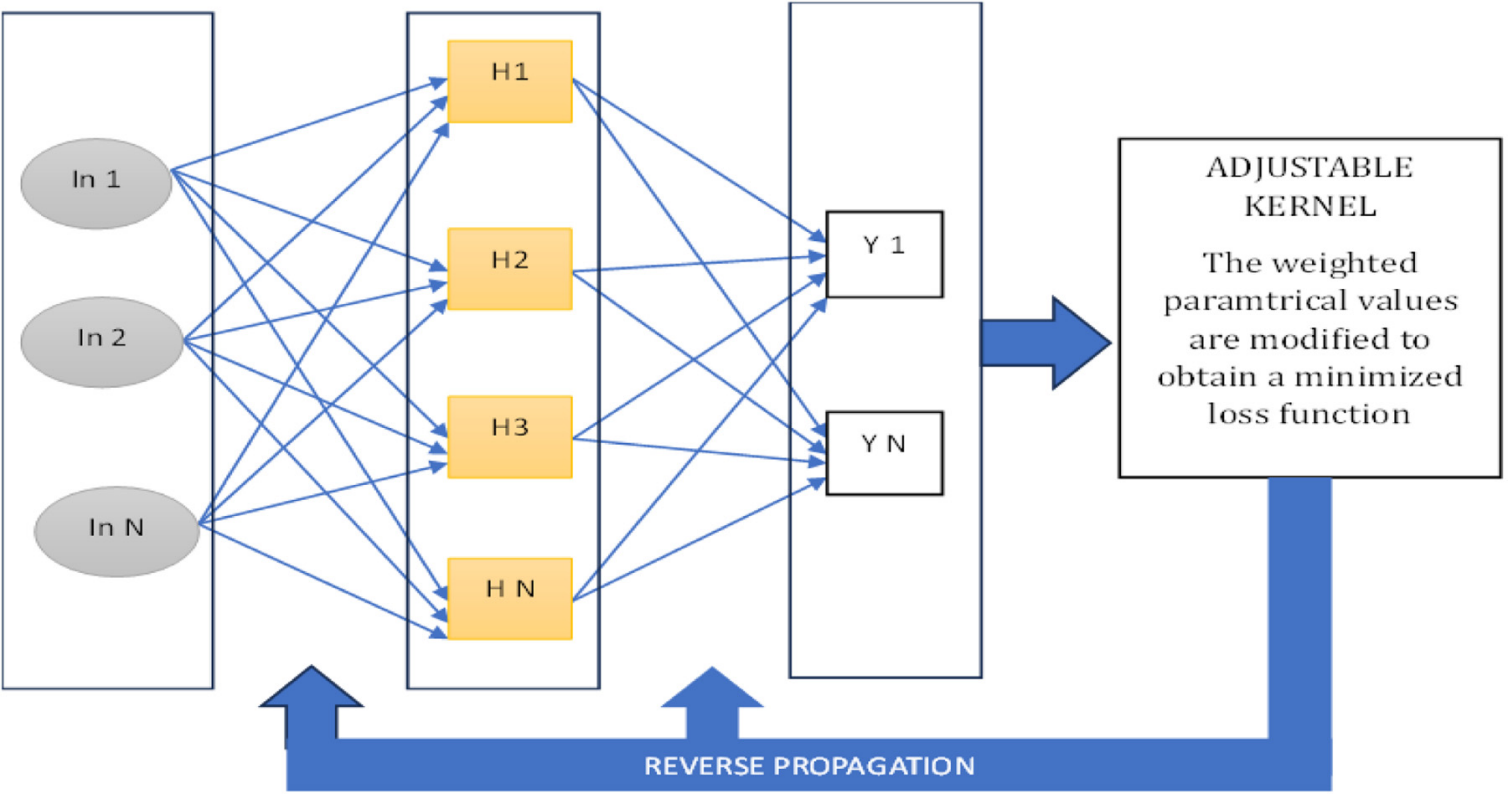
Temporal recurrent propagation unit.

Fig. 4 illustrates the temporal recurrent propagation process, comprising three distinct layers: input, hidden, and output. Every node in the hidden layer is entirely connected to each node in the input layer, enabling the transmission of information. The output layer is connected to the hidden layer, with the produced output routed to a configurable kernel. The weighted parameters are subsequently refined iteratively to reduce the loss function. Forward propagation entails the transmission of information from the input layer through the hidden layers to the output layer, whilst reverse propagation denotes the transmission of information from the kernel back via the hidden and input layers, facilitating the optimization of network parameters. This iterative method guarantees the effective learning and enhancement of the model.

Table 1 provides a detailed comparison of the Peak Signal-to-Noise Ratio (PSNR) between the proposed method and current methodologies. At the initial time step, the PSNR values for the repeat_P, repeat_F, SA_PFF, and TW_P_F techniques are 31, 25, 28, and 31, respectively. Conversely, the PSNR values for MC_Net, bi_SA, bi_TW, and the suggested BVPN_TA_RP technique are 34, 33, 35, and 37, respectively, demonstrating the latter's superior performance. The PSNR values for successive time steps are calculated and reported, further illustrating the improved efficacy of the suggested method throughout different stages of the video sequence.

**Table 1:** PSNR comparison of the proposed method with the existing methods.

Time steps	PSNR							
	Repeat_P	Repeat_F	SA_P_F	TW_P_F	MC_Net	bi_SA	bi_TW	BVPN_TA_RP
1	31	25	28	31	34	33	35	37
2	28	25	27	28	31	28	33	34
3	27	25	27	27	29	27	32	33
4	26	25	26	26	27	26	30	32
5	25	25	26	26	26	26	30	31
6	25	25	25	26	25	26	30	31
7	25	26	25	26	25	27	30	32
8	25	27	25	26	25	28	32	33
9	25	29	26	29	25	32	33	34
10	25	32	27	32	25	33	35	37

**Fig. 5:**
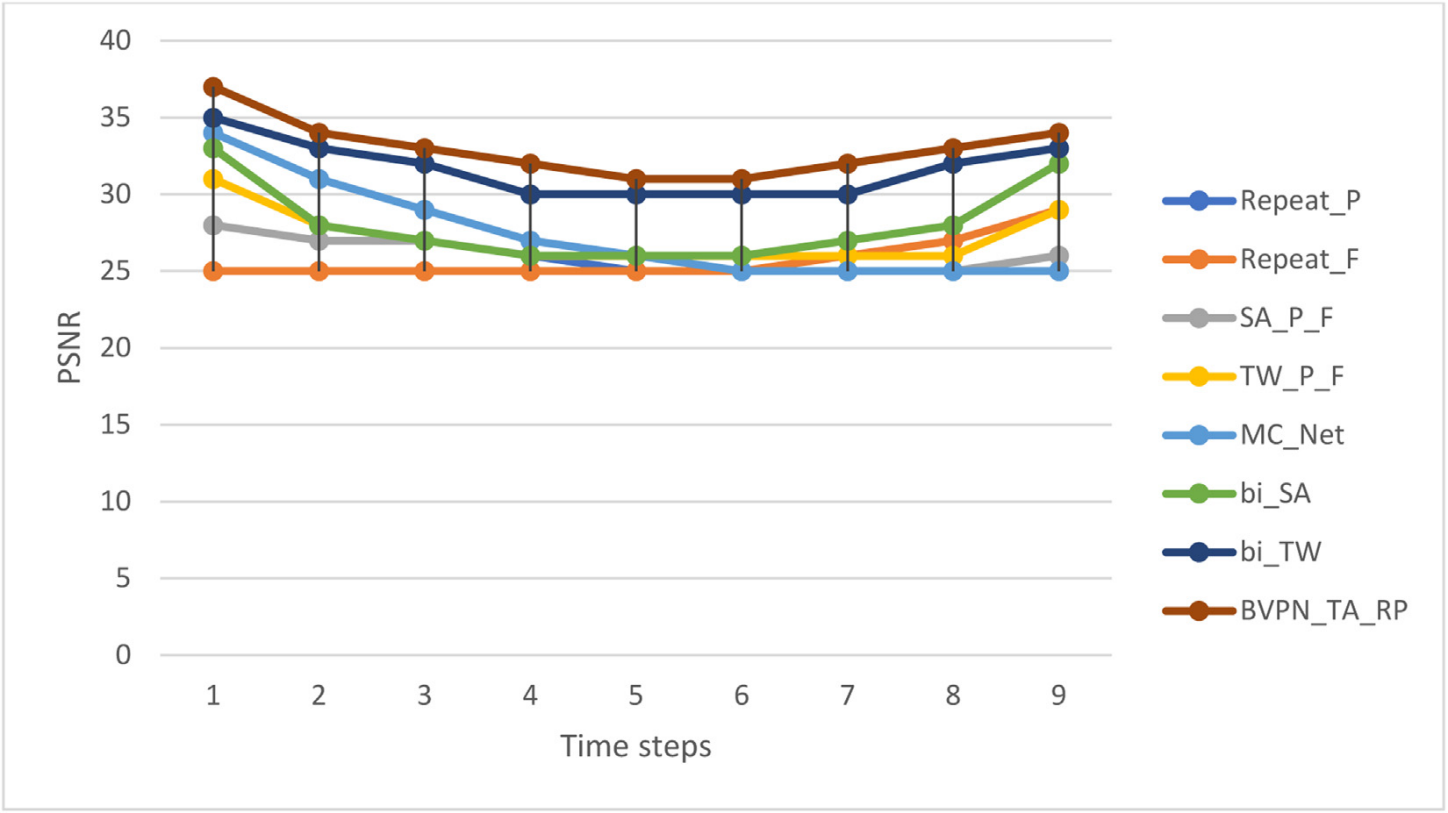
Graphical representation of the PSNRs values of the existing methods and the proposed method.

Fig. 5 illustrates the PSNR values, contrasting the efficacy of the existing methods with the newly proposed strategy. The graph effectively illustrates the enhanced performance of the suggested method at different time intervals, distinctly showcasing its elevated PSNR values compared to previous methods. This visual comparison highlights the efficacy of the proposed method for video frame quality reconstruction.

**Fig. 6:**
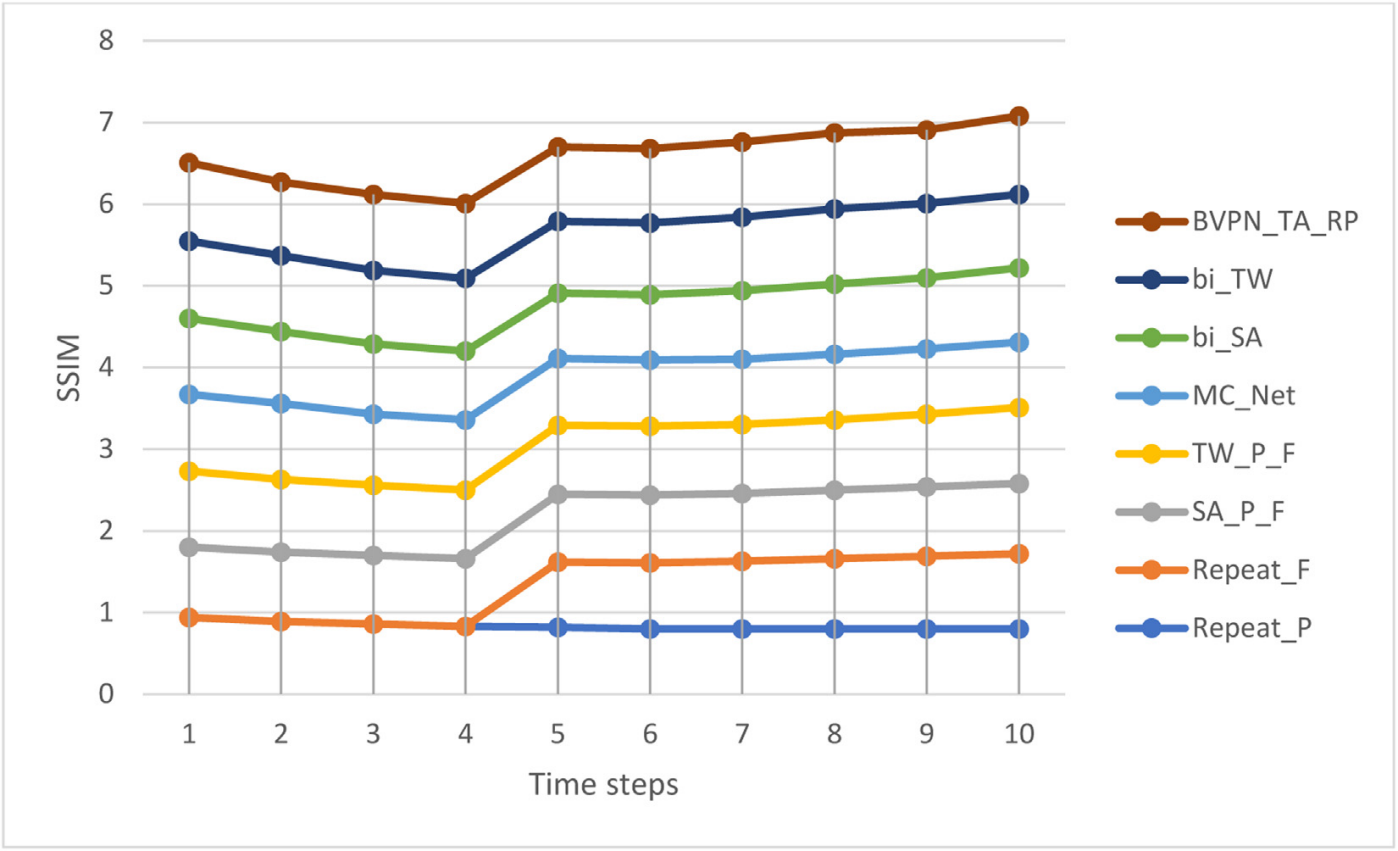
Graphical representation of SSIM values of the existing methods and the proposed method.

Table 2 illustrates a comparison of the SSIM values between the suggested approach and the established methods. Fig. 6 illustrates the SSIM values for both the established approaches and the newly proposed strategy, offering a clear graphical depiction of their performance. The comparison underscores the enhancement in structural similarity attained by the suggested method, demonstrating its superior capacity to preserve the structural integrity of video frames relative to previous methodologies.

**Table 2:** SSIM comparison of the proposed method with the existing methods.

Time steps	SSIM							
	RepeatP	Repeat_F	SA_P_F	TW_P_F	MC_Net	bi_SA	bi_TW	BVPN_TA_RP
1	0.94	0.80	0.86	0.93	0.94	0.93	0.95	0.96
2	0.89	0.80	0.85	0.89	0.93	0.88	0.93	0.90
3	0.86	0.80	0.84	0.86	0.87	0.86	0.90	0.93
4	0.83	0.80	0.83	0.84	0.86	0.84	0.89	0.92
5	0.82	0.80	0.83	0.84	0.82	0.80	0.88	0.91
6	0.80	0.81	0.83	0.84	0.81	0.80	0.88	0.91
7	0.80	0.83	0.83	0.84	0.80	0.84	0.90	0.92
8	0.80	0.86	0.84	0.86	0.80	0.86	0.92	0.93
9	0.80	0.89	0.85	0.89	0.80	0.87	0.91	0.90
10	0.80	0.92	0.86	0.93	0.80	0.91	0.90	0.96

**Fig. 7:**
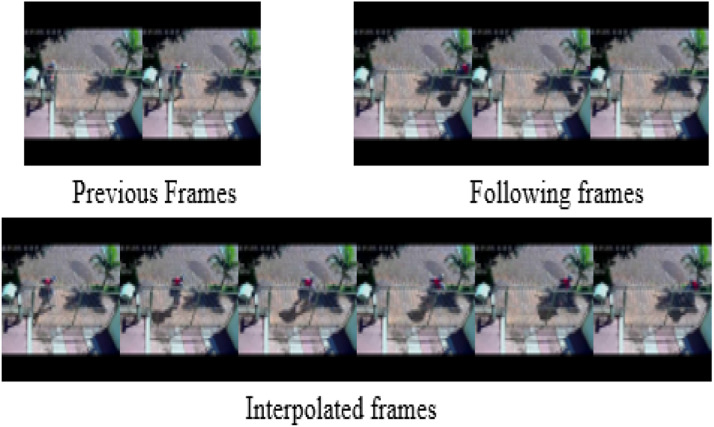
Previous, Following and Interpolated frames of a video sequence.

Fig. 7 displays images from the video interpolation tasks, illustrating the efficacy of the suggested method in inpainting video frames. In contrast to current video inpainting methods, which frequently encounter difficulties in achieving seamless frame reconstruction, our technique successfully retrieves whole and continuous frames. Unlike frame interpolation and video prediction techniques that depend on projecting future frames based on previous information, our approach forecasts the desired sequence by employing several frames that are simultaneously present before and after the absent frames. Fig. 7 illustrates that the frames preceding (0, 1) and following (8, 9, 10) the missing sequence are utilized to rebuild the interpolated frames, which are presumably absent (2, 3, 4, 5, 6, 7). Table 3 demonstrates the fluctuation in PSNR values for varying quantities of input frames, emphasizing the influence of many frames on the efficacy of the video inpainting procedure.

**Table 3:** Variation in the PSNR with different numbers of input frames.

Time Steps	PSNR			
	BVPN_TA_RP with 2 input frames	BVPN_TA_RP with 3 input frames	BVPN_TA_RP with 4 input frames	BVPN_TA_RP with 5 input frames
1	35.3	35.5	36.6	36.7
2	32.1	32.3	33.4	33.6
3	30.2	30.6	31.7	31.9
4	29.1	29.4	30.5	30.6
5	28.3	28.6	29.7	29.9
6	29.4	29.5	30.5	30.8
7	29.8	29.9	30.1	30.3
8	31.2	31.5	32.6	33.8
9	32.2	32.6	33.7	33.9
10	35.3	35.6	36.6	36.8

**Fig. 8:**
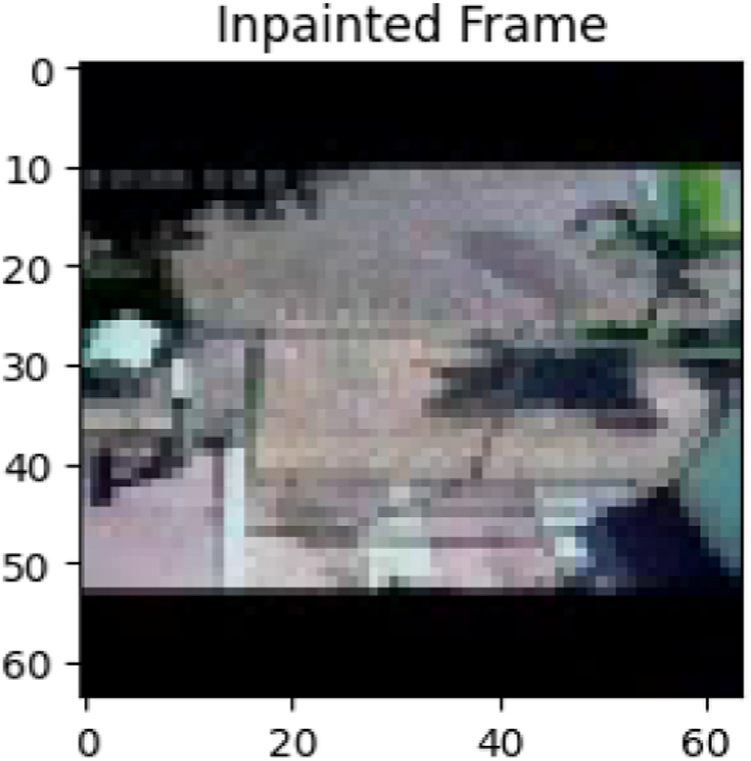
Inpainted frame.

**Fig. 9:**
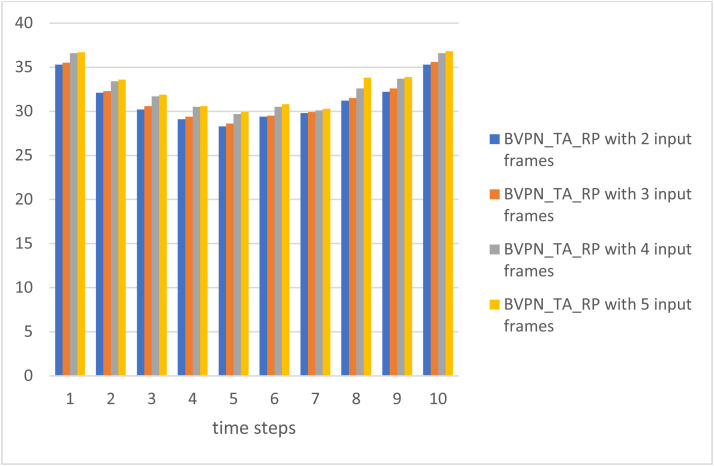
Graphical representation of the variation in PSNR with different numbers of input frames.

**Fig. 10:**
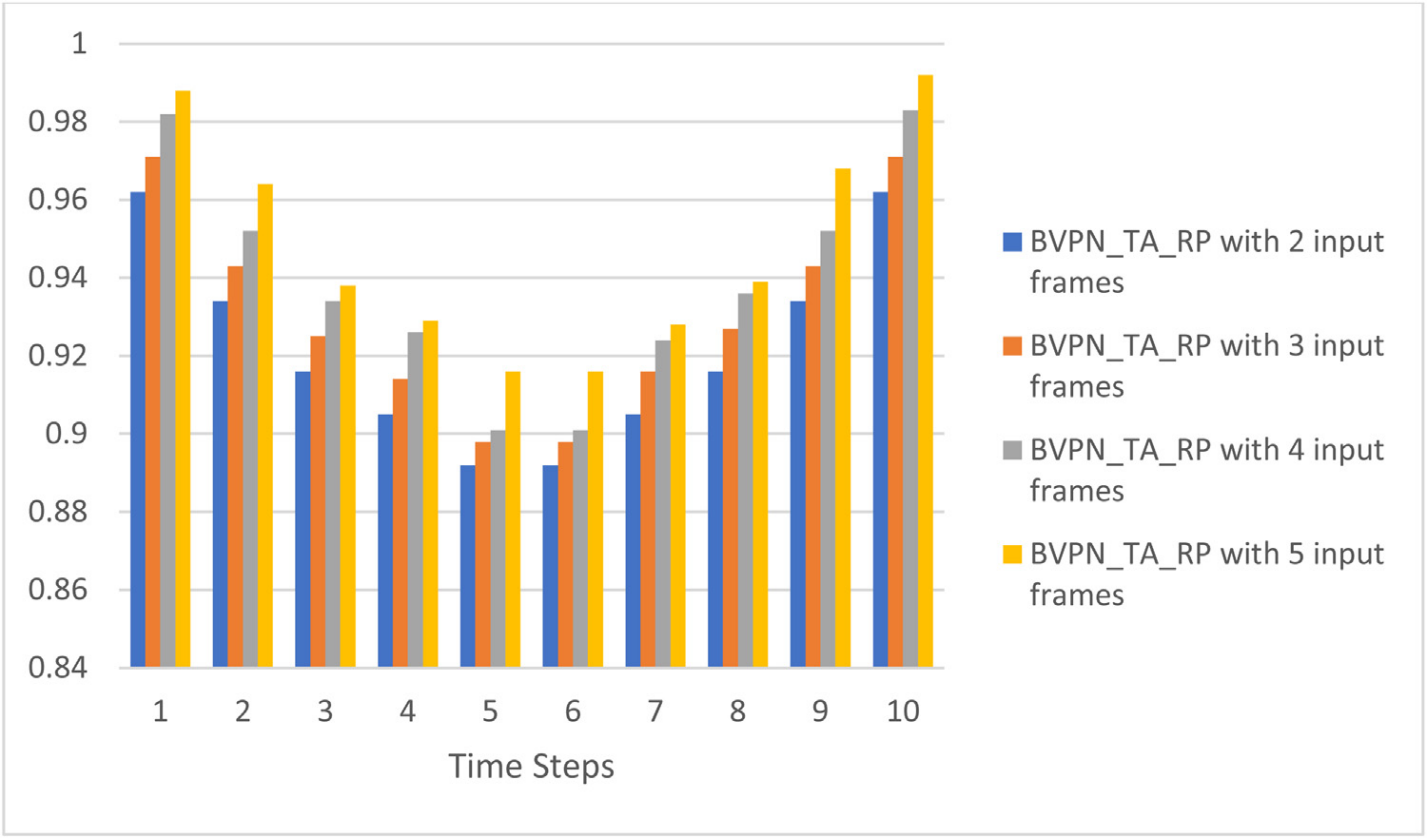
Graphical representation of the variation in SSIM with different numbers of input frame.

Fig. 8 shows the inpainted interpolated frame, showcasing the efficacy of the suggested method in flawlessly reconstructing absent frames. Table 4 illustrates the fluctuation in SSIM values corresponding to different quantities of input frames, emphasizing the influence of supplementary input frames on the structural similarity between the original and predicted frames. Fig. 9 illustrates the correlation between PSNR and the quantity of input frames, indicating that an increase in PSNR corresponds to enhanced image quality. The bidirectional video prediction network (BVPN_TA_RP), employing temporal aggregation and recurrent propagation with five input frames, attains exceptional performance. Furthermore, Fig. 10 illustrates the fluctuation in SSIM values corresponding to varying quantities of input frames, so highlighting the enhanced performance of the BVPN_TA_RP model regarding structural similarity.

**Table 4:** Variation in SSIM with different numbers of input frames.

Time Steps	SSIM			
	BVPN_TA_RP with 2 input frames	BVPN_TA_RP with 3 input frames	BVPN_TA_RP with 4 input frames	BVPN_TA_RP with 5 input frames
1	0.962	0.971	0.982	0.988
2	0.934	0.943	0.952	0.964
3	0.916	0.925	0.934	0.938
4	0.905	0.914	0.926	0.929
5	0.892	0.898	0.901	0.916
6	0.892	0.898	0.901	0.916
7	0.905	0.916	0.924	0.928
8	0.916	0.927	0.936	0.939
9	0.934	0.943	0.952	0.968
10	0.962	0.971	0.983	0.992

## Conclusion

The suggested technique efficiently reconstructs absent chunks of a video by utilizing the available frames. It utilizes a bidirectional video prediction network, temporal aggregation, and recurrent propagation to analyse and forecast the absent intermediate frames between the existing input frames. The bidirectional video prediction network interprets intermediate frames, temporal aggregation employs available information to generate the goal frame, and recurrent propagation preserves temporal consistency by reusing prior data. The comparative comparison with existing methods indicates that the suggested model surpasses others, as seen by the elevated PSNR and SSIM values achieved for the UCF-Crime dataset, which are displayed in both tabular and graphical formats. This research offers significant insights for enhancing frame prediction approaches across diverse video applications, establishing a basis for further investigation in this field.

## Limitations

Not applicable.

## Ethics statements

The paper reflects the authors' own research and analysis in a truthful and complete manner.

## CRediT author statement

**Mohana Priya:** Conceptualization, Methodology, Software, Writing- Original draft preparation, Conceptualization, Investigation, **UlagaPriya K:** Visualization, Data curation, Investigation, Supervision, Data curation, Investigation.

## Declaration of competing interests

The authors declare that they have no known competing financial interests or personal relationships that could have appeared to influence the work reported in this paper.

## Data Availability

The datasets analysed during the current study are available in the weblink repositoryhttps://www.kaggle.com/datasets/odins0n/ucf-crime-dataset.
